# Alterations of Subcortical Brain Structures in Paradoxical and Psychophysiological Insomnia Disorder

**DOI:** 10.3389/fpsyt.2021.661286

**Published:** 2021-05-07

**Authors:** Farnoosh Emamian, Mostafa Mahdipour, Khadijeh Noori, Masoumeh Rostampour, S. Bentolhoda Mousavi, Habibolah Khazaie, Mohammadreza Khodaie-Ardakani, Masoud Tahmasian, Mojtaba Zarei

**Affiliations:** ^1^Department of Psychiatry, University of Social Welfare and Rehabilitation Sciences, Tehran, Iran; ^2^Sleep Disorders Research Center, Kermanshah University of Medical Sciences, Kermanshah, Iran; ^3^Institute of Medical Science and Technology, Shahid Beheshti University, Tehran, Iran; ^4^Psychosis Research Center, University of Social Welfare and Rehabilitation Sciences, Tehran, Iran

**Keywords:** insomnia disorder, paradoxical insomnia, psychophysiological insomnia, shape analysis, gray matter volume, subcortical brain structures

## Abstract

Insomnia disorder (ID) is a common illness associated with mood and cognitive impairments. Subtyping ID is an ongoing debate in sleep medicine, but the underlying mechanisms of each subtype is poorly understood. Growing evidence suggests that subcortical brain structures play the key roles in pathophysiology of ID and its subtypes. Here, we aimed to investigate structural alteration of subcortical regions in patients with two common ID subtypes i.e., paradoxical and psychophysiological insomnia. Fifty-five patients and 49 healthy controls were recruited for this study and T1-weighted images and subjective and objective sleep parameters (i.e., Pittsburgh Sleep Quality Index and polysomnography) were collected from participants. Subcortical structures including the hippocampus, amygdala, caudate, putamen, globus pallidus, nucleus accumbens, and thalamus were automatically segmented in FSL. Volume and shape (using surface vertices) of each structure were compared between the groups, controlled for covariates, and corrected for multiple comparisons. In addition, correlations of sleep parameters and surface vertices or volumes were calculated. The caudate's volume was smaller in patients than controls. Compared with controls, we found regional shrinkage in the caudate, nucleus accumbens, posterior putamen, hippocampus, thalamus, and amygdala in paradoxical insomnia and shrinkage in the amygdala, caudate, hippocampus, and putamen in psychophysiological insomnia. Interestingly, comparing two patients groups, shape alteration in the caudate, putamen, and nucleus accumbens in paradoxical insomnia and shrinkage in the thalamus, amygdala, and hippocampus in psychophysiological insomnia were observed. Both subjective and objective sleep parameters were associated with these regional shape alterations in patients. Our results support the differential role of subcortical brain structures in pathophysiology of paradoxical and psychophysiological insomnia.

## Introduction

Insomnia disorder (ID) is characterized by problems in initiating or maintaining sleep, or early morning awakening. The daytime consequences are fatigue, mood disturbance, and cognitive impairment (1, 2). Definition of chronic ID, based on the third edition of International Classification of Sleep Disorders (ICSD-3) criteria (3, 4), requires insomnia symptoms to occur for at least three times per week and lasts for more than 3 months. Rising prevalence of ID (3.9–22.1%) is probably due to genetic and psychosocial factors including aging population, high level of stress, and increasing rate of depression and anxiety in the modern societies (1). In addition to a significant economic burden (5), ID is associated with elevated body-mass index (BMI), higher rate of cardiovascular diseases, increased amount of motor vehicle accidents, and various psychiatric comorbidities such as depression (6–11). Despite all the severe medical and mental consequences of ID, its pathophysiology is poorly understood.

Several neuroimaging studies revealed widespread structural and functional cortical changes (12–14) including gray matter atrophy in the orbitofrontal cortex, dorsolateral prefrontal, pericentral cortices, temporal cortex, and precuneus, but increased gray matter volume in the anterior cingulate cortex (ACC). For review see (13, 14). The role of subcortical brain regions in pathophysiology of ID was previously examined, although the results were inconsistent and difficult to replicate. For review see (13, 14). A surface-based shape analysis of 27 patients with ID revealed that poor sleep quality and higher arousal are associated with subcortical atrophy including the hippocampus, amygdala, basal ganglia, and thalamus that was linked with impaired cognitive functions (15). Gong et al. also found regional atrophy in the amygdala, which was related to the severity of insomnia and anxiety in ID patients (16). Moreover, the critical role of amygdala toward negative sleep-related stimuli (17), the role of hippocampus on sleep-related maladaptive rumination (18, 19), and the role of caudate on hyperarousal state of patients (20) have been observed in ID previously. These studies collectively point to an important role of subcortical brain regions in pathophysiology of ID. Recently, we performed a neuroimaging meta-analysis on 19 ID studies, but failed to identify convergent regional abnormality (21). This indicates that ID heterogeneity is not only due to the variant neuroimaging data acquisition and analysis methods, but also related to clinical variability of the patients e.g., including different ID subtypes (22, 23). Thus, there is a clear need for more detailed investigations of the brain structures on the well-characterized subtypes of ID.

The second version of ICSD introduced several ID subtypes such as paradoxical insomnia and psychophysiological insomnia (24). Paradoxical insomnia is characterized by subjective sleep loss and daily insomnia symptoms, but normal objective sleep profile [e.g., using polysomnography (PSG)], indicating a discrepancy between subjective and objective sleep patterns (25). On the other hand, psychophysiological insomnia is characterized by the “learned sleep-preventing association,” which indicates that pre-sleep condition appears to be classically conditioned to the bedroom environment and prevents sleep (26). Hence, paradoxical insomnia is defined by misperception of sleep, while psychophysiological insomnia is characterized by fear of sleep and bedroom environment. The ICSD-3 highlights that physiological abnormalities in sleep tracing are present in various subtypes, but they are often subtle and could not be detected by available routine sleep recording methods and hence, subtyping ID should be ignored in clinical practice (3). However, several studies support subtyping ID, using various neurophysiological, cognitive and psychological methods (22, 25, 27, 28). Recently, Blanken et al. applied a data-driven approach on a multidimensional set of biologically based traits in a large-scale population and identified five new ID subtypes (29). This study further suggests that ID should be considered as a heterogenic disorder (22) and subtyping may resolve inconsistencies to identify differential etiologies of ID (29).

In the present study, we explored possible structural alterations in the subcortical gray matter structure (i.e., the caudate, putamen, pallidum, nucleus accumbens, thalamus, amygdala, and hippocampus) using volume and shape analyses based on surface vertices. Our main question was whether there is any structural difference between two main ID subtypes and whether these changes are associated with insomnia symptoms. It was assumed that there are more structural alterations in the areas responsible for sleep perception and regulation of sleep-wake patterns in patients with paradoxical insomnia, while there are more changes in the regions responsible for sleep-related anxiety and hyperarousal in patients with psychophysiological insomnia.

## Methods

### Subjects

We recruited 116 participants in the study. Chronic ID patients were recruited from Sleep Disorders Research Center, Kermanshah University of Medical Sciences. All patients were interviewed by a sleep specialist (H.K.) and met diagnostic criteria of ID according to ICSD and psychiatric interview, overnight PSG, and Pittsburgh Sleep Quality Index (PSQI) before brain MRI acquisition. Healthy subjects were recruited through local advertisement and were defined as those with no neurological or psychiatric illness at present or past and total PSQI score <5. Our exclusion criteria included taking any neuropsychiatric medications, pregnancy, any other medical, neurological, or psychiatric conditions, as well as contraindications to MR imaging. The study was approved by Ethics Committee of Kermanshah University of Medical Sciences and written informed consent was obtained from all participants. Two patients with comorbid periodic leg movement, five patients with mild/moderate obstructive sleep apnea, one patient with hydrocephaly, two patients with brain mass, and two subjects with too much movements in the scanner (which caused distortion in the images) were excluded from the study. Finally, analyses were performed on 55 chronic ID patients (including 29 individuals with paradoxical insomnia and 26 patients with psychophysiological insomnia), as well as 49 healthy subjects.

### Insomnia Assessments

The patients were asked to avoid taking coffee, tea, heavy diet, and smoking during the day of the experiment. Our imaging assessment was carried out when the patients stopped taking any hypnotic medication for at least a week before imaging assessment. We excluded any patient, who was addicted to hypnotic medications such as benzodiazepines. The subjects arrived to the sleep center at 9 pm and completed the demographic and PSQI questionnaires. PSG measurements using SOMNOscreen™ plus model (Somnomedics, Germany) were performed at least 7 h based on the usual sleeping habits of subjects. Sleeping room was standardized for any noise and visual stimulus based on the international protocols (30). Diagnosis of ID subtypes was mainly based on ICSD-2 (24). All patients met diagnostic criteria for chronic ID based on ICSD-3 as well (3, 4), which are largely congruent with ICSD-2. They include a subjective report of sleep initiation or maintenance problems, adequate opportunity to sleep, as well as daytime consequences. In addition, insomnia symptoms were presented for at least three times per week and lasts for more than 3 months in our chronic ID groups. The insomnia symptoms were not associated with substance abuse and other psychiatric or sleep disorders. Paradoxical insomnia was diagnosed by the complaints of short sleep duration and poor sleep quality despite near-normal objective sleep patterns in PSG i.e., the misperception index ≥ 0.9 (31). Detailed criteria for paradoxical insomnia diagnosis include (i) subjective insomnia symptoms, but total sleep time (TST) > 6 h and 30 min and sleep efficiency (SE) > 85% using overnight PSG; (ii) discrepancy between objective (PSG) and subjective (self-report) sleep measures (i.e., a difference of 60 min or more for TST, or a difference of at least 15% for SE) (32). Psychophysiological insomnia were defined based on psychiatric interview, subjective insomnia symptoms, as well TST <6 h and 30 min and SE <85% (24), indicating that subjective and objective sleep assessment parameters are congruent in patients with psychophysiological insomnia. There is no difference on sleep quality, assessed by PSQI questionnaires, between the patients groups (*p* > 0.05) (Table 1).

**Table 1:** Demographics and clinical characteristics of all participants.

**Variables**	**Paradoxical insomnia (*n* = 29)**	**Psychophysiological insomnia (*n* = 26)**	**Controls (*n* = 49)**	***P*-value (Paradoxical vs. Controls)**	***P*-value (Psychophysiological vs. Controls)**	***P*-value (Psychophysiological vs. Paradoxical)**
Age (years)	43.76 ± 10.78	47.15 ± 12.08	38.92 ± 12.1	0.079	0.006	0.27
Gender (Male: Female)	10:19	13:13	20:29	0.51	0.51	0.25
Sleep quality (total PSQI score)	15.93 ± 2.72	15.88 ± 3.10	2.96 ± 1.59	0.00	0.00	0.95
Disease duration (years)	6.69 ± 6.75	9.3 ± 9.35	-	-	-	0.23
Total sleep time (min)	420.21 ± 37.28	299.81 ± 101.83	-	-	-	0.00
Sleep efficiency (%)	87.58 ± 7.4	63.1 ± 21.32	-	-	-	0.00

*Data are presented as mean ± SD*.

*PSQI, Pittsburgh sleep quality index*.

### MRI Acquisition and Quality Control

MRI was performed using a whole-body 1.5T Siemens Magnetom Avanto scanner with an 8- channel head coil. Structural images were acquired with a high-resolution, T1-weighted MPRAGE (TR = 1,950 ms, TE = 3.1 ms, flip angle = 15°, FOV = 256 × 256 mm^2^, matrix = 256 × 256 mm^2^, voxel size = 1 × 1 × 1 mm^3^, 176 sagittal slices). All images were visually checked by a radiologist to rule out any gross brain pathology. Quality control of data was carried out using the University of Southern California quality assurance pipeline (https://qc.loni.usc.edu/).

### Segmentation of Subcortical Structures and Shape and Volume Analyses

The FIRST tool (part of FMRIB Software Library) was used to automatically segment seven subcortical brain structures including the caudate, putamen, pallidum, nucleus accumbens, thalamus, amygdala, and hippocampus in each hemisphere (33, 34). In brief, the FIRST is a probabilistic adaptation of the active appearance model. The method is informed by the shape and intensity variations of a structure from a training set for the purpose of automatically segmenting the structures. Surface of each structure is modeled by a deformable mesh, composed of a set of triangles and vertices. There are a fixed number of vertices with arbitrary positions for each structure. A multivariate Gaussian model of vertex location and intensity variation is used and is based on having point correspondence across subjects (same number and labeling of vertices across subjects). The necessary correspondence is imposed during the parameterization of the labeled images with a deformable model. The model is fit to new images by maximizing the posterior probability of shape given the observed intensities (33).

Between groups shape comparisons were carried out by comparing coordinates of each corresponding vertex, i.e., vertex-wise analysis, described in the FIRST tool (33, 34). We used the Randomize (part of FSL), which is a non-parametric permutation testing (10,000 permutations in our study) and allows modeling and inference using standard general linear model (GLM) design (35). We also compared the mean volume of subcortical regions between groups using GLM design in the FIRST. For both shape and volume analyses, we controlled for age, gender, and total brain volume as covariates of no-interest. For shape analysis, we used threshold-free cluster enhancement (TFCE) correction (36) to avoid arbitrary thresholds selection. Correction for multiple comparisons was applied using false discovery rate (FDR) correction (37). In patients, association between vertices and total PSQI/sleep efficiency was assessed using Spearman's rho two-tailed tests, with correction for covariates of no-interest (i.e., age, gender, and total brain volume) and multiple comparisons using FDR correction. Finally, for volume comparison, we used Least Significant Difference adjustment for multiple comparisons.

## Results

### Clinical Findings

Demographic data are presented in Table 1. No statistical differences were found for age (*p* = 0.079) or gender (*p* = 0.51) between healthy controls and paradoxical insomnia patients, while there were significant differences in age (*p* = 0.006), but not in gender (*p* = 0.51) between healthy controls and psychophysiological insomnia patients. The paradoxical and psychophysiological insomnia groups had no significant differences regarding age (*p* = 0.27) and gender (*p* = 0.25). In addition, paradoxical (*p* = 0.000) and psychophysiological (*p* = 0.000) insomnia patients had higher PSQI scores (poor sleep quality) than healthy controls, without any significant difference between two ID groups (*p* = 0.95). As expected, TST (*p* = 0.000) and SE (*p* = 0.000) were significantly better in paradoxical vs. psychophysiological insomnia patients. No statistical differences were found for duration of disease (*p* = 0.23) between two ID subtypes. Regarding total brain volume, healthy control subjects (1569.4 ± 22.5 cm^3^) had significantly larger brains than paradoxical (1447.3 ± 30.8 cm^3^) or psychophysiological insomnia groups (1485.7 ± 33.0 cm^3^) with *p*-values of 0.001 and 0.04, respectively. There was no significant difference between total brain volume of paradoxical and psychophysiological insomnia patients (*p* = 0.3).

### Volumetric Findings of Subcortical Structures

Mean volume of the left caudate was significantly smaller in the patient with paradoxical insomnia compared to psychophysiological insomnia, as well as to the control group. In addition, volume of bilateral caudate was different between paradoxical insomnia and psychophysiological insomnia (Table 2).

**Table 2:** Volume of subcortical structures (mm3). Data are presented as mean ± SD.

**Structure**	**Healthy controls**	**Paradoxical insomnia**	**Psychophysiological insomnia**
Left accumbens	832 ± 134	791 ± 119	794 ± 166
Left amygdala	1845 ± 288	1616 ± 335	1805 ± 268
Left caudate*^$^	4804 ± 535	4654 ± 655	4627 ± 390
Left hippocampus	5074 ± 438	4760 ± 518	5129 ± 553
Left pallidum	1840 ± 198	1758 ± 180	1845 ± 220
Left putamen	5259 ± 547	4967 ± 516	5157 ± 494
Left thalamus	8086 ± 706	7462 ± 757	8006 ± 706
Right accumbens	679 ± 127	633 ± 131	650 ± 103
Right amygdala	1805 ± 312	1725 ± 295	1705 ± 300
Right caudate^#^	4937 ± 565	4758 ± 559	4685 ± 406
Right hippocampus	5160 ± 434	4959 ± 455	5307 ± 422
Right pallidum	1827 ± 193	1736 ± 189	1831 ± 152
Right putamen	5226 ± 553	4958 ± 526	5085 ± 510
Right thalamus	7809 ± 681	7265 ± 739	7823 ± 643

* *Comparing control with paradoxical insomnia (F = 6.273, P = 0.014)*.

$ *Comparing paradoxical with psychophysiological insomnia (F = 4.666, P = 0.035)*.

# *Comparing paradoxical with psychophysiological insomnia (F = 7.592, P = 0.008)*.

### Vertex-Wise Shape Analysis Findings

**Figure 1:**
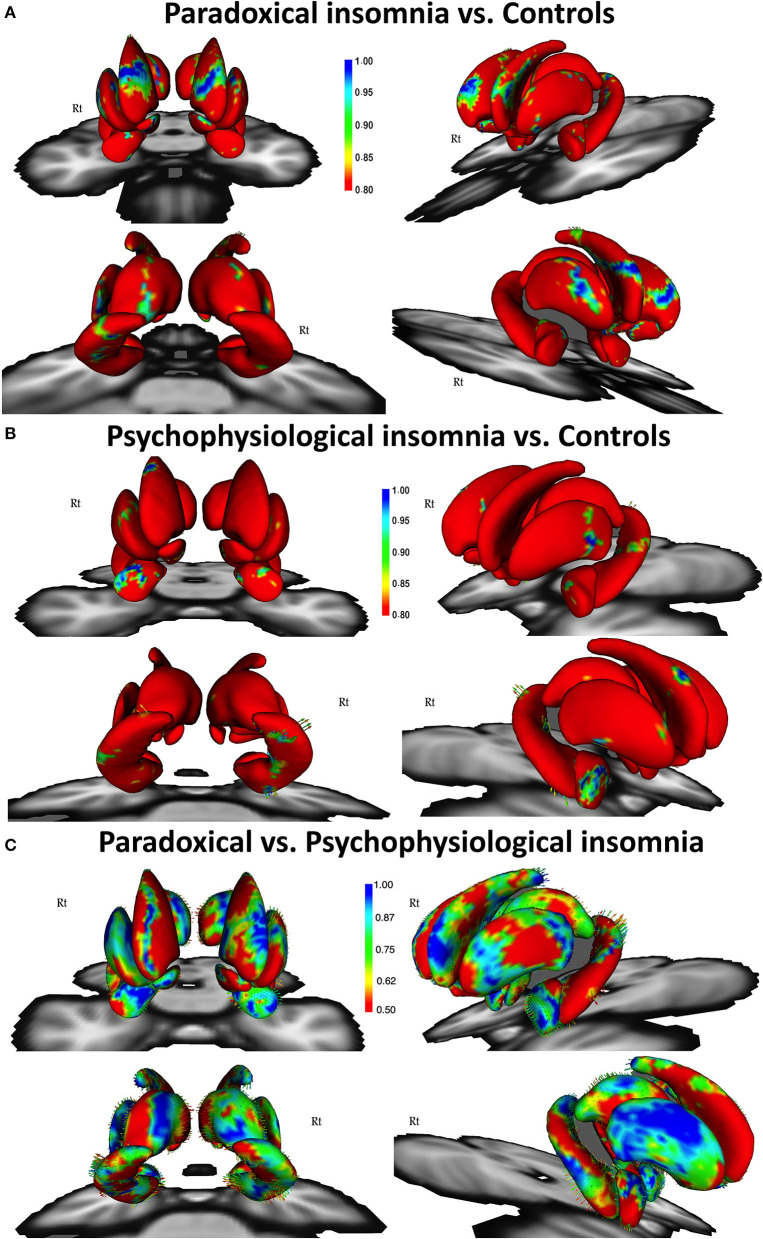
Vertex-wise surface analysis comparing patients with paradoxical insomnia vs. control group **(A)**, patients with psychophysiological insomnia vs. control group **(B)**, patients with paradoxical insomnia vs. psychophysiological insomnia **(C)** including covariates of no-interest (i.e., age, gender, and total brain volume). Color bar shows false discovery rate (FDR) corrected *p*-values.

Comparing paradoxical insomnia with healthy subjects, we found alterations in the anterior dorsal caudate, tail of left hippocampus, dorsal anterior thalamus, superior part of right amygdala, and anterolateral part of left putamen (*p* < 0.05, FDR corrected) (Figure 1A). Comparing psychophysiological insomnia with control individuals, we observed shrinkage in the anterior amygdala, dorsal part of right caudate, head and body of hippocampus (left tail > right tail), lateral part of putamen (right tail > left tail) (*p* < 0.05, FDR correction) (Figure 1B). Moreover, comparing two ID groups showed that bilateral hippocampus (head, ventral aspect of the body and dorsal aspect of the tail), dorsal thalamus, and anterior amygdala were shrunk in psychophysiological insomnia compared to paradoxical insomnia. In contrast, the caudate, putamen and nucleus accumbens (mainly the right side) showed relative shrinkage in paradoxical insomnia than psychophysiological insomnia (*p* < 0.05, FDR correction) (Figure 1C).

### Association Between Shape Abnormalities and Sleep Parameters

**Figure 2:**
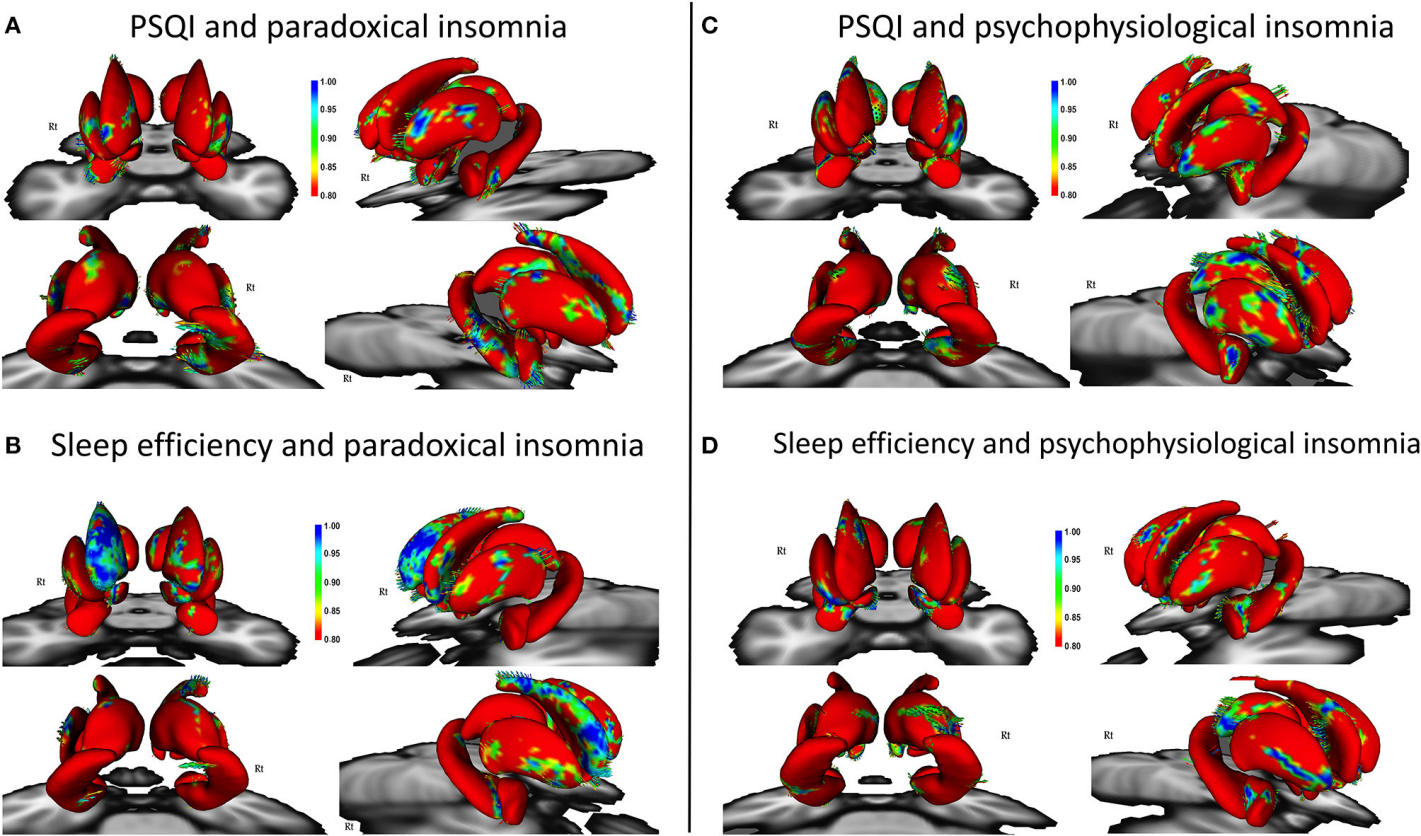
Correlation of Pittsburgh Sleep Quality Index (PSQI) and sleep efficiency with surface changes in patients with paradoxical insomnia **(A,B)**; Correlation of PSQI and sleep efficiency with surface changes in patients with psychophysiological insomnia **(C,D)** including covariates of no-interest (i.e., age, gender, and total brain volume). Color bar shows false discovery rate (FDR) corrected *p*-values.

Patients with paradoxical insomnia, showed positive correlations between PSQI and shape changes in the putamen, nucleus accumbens, head and body of hippocampus (right tail > left tail), and anterior and posterior extremes of the caudate, but negative correlation with body of right caudate (Figure 2A). SE negatively correlated with dorsal and ventral part of the caudate, but positively correlated with head and tail of the caudate (right > left), nucleus accumbens, and posterior putamen (Figure 2B).

In patients with psychophysiological insomnia, PSQI scores were negatively correlated with the anterior amygdala, head and tail of caudate, head and body of hippocampus, and posterior dorsal thalamus, but positively correlated with the lateral part of putamen (Figure 2C). In addition, SE was positively correlated with shape changes in the anterior amygdala, right dorsal putamen and caudate, as well as inferior part of the hippocampus and posterior dorsal left thalamus, but negatively correlated with lateral part of the putamen (Figure 2D).

## Discussion

ID is clinically a heterogeneous disorder and current ID literature indicates remarkable inconsistencies in terms of clinical features and treatment response (22, 29), which suggests that a bottom-up classification of ID should be reconsidered in sleep medicine (22, 25). Recently, a data-driven approach using multivariate profiles of affect, personality, and life history of 2,224 participants with ID and 2,098 controls was performed (29). Their result identified five new ID subtypes including highly distressed, moderately distressed but reward sensitive, moderately distressed and reward insensitive, slightly distressed with high reactivity, and slightly distressed with low reactivity (29). Previously, Turcotte et al. using event-related potentials measures demonstrated that psychophysiological insomnia had inability to inhibit information processing during sleep onset, but paradoxical insomnia showed enhanced attentional processing, which results in a higher need for inhibition (38). The present study suggests structural difference, not only between ID and healthy subjects, but also between paradoxical and psychophysiological insomnia, indicating importance of previously suggested subtypes in ICSD-2 for ID. In particular, we demonstrated that subcortical brain areas of patients with two ID subtypes undergo different structural alterations, and these changes are associated with both subjective and objective sleep-related measures.

Given the fact that the caudate nucleus showed atrophy and shrinkage in paradoxical insomnia (and not in psychophysiological insomnia), our shape and volumetric findings indicate a critical role of caudate in subjective-objective sleep discrepancy in paradoxical insomnia (25, 27, 39). A typical patient with paradoxical insomnia often feels that s/he has not slept enough, but polysomnographic data shows normal sleep duration. Our notion is in support of a crucial function of the caudate in pathophysiology of ID (20), particularly those with paradoxical insomnia who have sleep-state misperception (39). There is considerable evidence that the striatum (including caudate and putamen) plays an important role in sleep behavior. The caudate receives input from the orbitofrontal and parietal cortices, and the putamen receives input from the somatosensory, primary motor, and premotor cortices (40). Structural alterations in the cortically connected area such as the orbitofrontal cortex to the caudate has been reported previously in ID (13). The basal ganglia are recognized for disparate functions not only regulating movements, cognitive, affective and somatosensory functions, but also regulating sleep-wake patterns (40, 41). Indeed, dorsal striatum augments wakefulness and nucleus accumbens regulates sleep-wake pattern by promoting sleep (41). Sleep disturbances contribute to the striatal dopamine levels too. Adenosine and dopamine receptors in the ventral striatum promote wakefulness by motivational behavior, but locomotor and arousal systems are inhibited during sleep (41). Stoffers et al. demonstrated an impaired recruitment of the head of left caudate nucleus during executive functioning, which was associated with hyperarousal severity in ID patients (20). This may be predispose and perpetuate hyperarousal and insomnia. However, cognitive behavioral therapy for insomnia (CBT-I) did not normalize the observed hypoactivation of the caudate during the executive task performance (20). Literature is lacking for the specific role of the putamen in ID, but shrinkage in the putamen in obstructive sleep apnea and rapid eye movement (REM) sleep behavior disorder, and also increased synaptic dopamine in putamen in restless legs syndrome have been reported earlier (42–44). These findings collectively point out to the critical role of the striatum including the caudate and putamen in pathophysiology of ID, particularly paradoxical insomnia.

On the other hand, patients with psychophysiological insomnia revealed that poor subjective sleep quality (i.e., higher total PSQI score) was associated with regional shrinkage of thalamus, amygdala, hippocampus, putamen and caudate. In addition, low sleep efficiency was linked with shrinkage in the thalamus, amygdala, hippocampus, caudate, and dorsal putamen, as well as hypertrophy in the right posterior thalamus. Comparing two patients groups showed that although various subcortical regions undergo structural changes in psychophysiological insomnia, it is mainly associated with shrinkage in thalamus, amygdala, and hippocampus. This is in line with the hyperarousal model of insomnia and emotional memory impairment in ID (14, 45). This model suggests that ID is characterized by increased arousal at the physiological, endocrine, cognitive or emotional levels and increased amygdala activity (17). Importantly, poor subjective and objective sleep quality is linked with enlargement of lateral putamen, which might be a compensatory mechanism to dysfunction of the limbic circuits. Similar to our findings, Koo et al. found an association between subcortical atrophic shape abnormalities in the thalamus, amygdala, hippocampus, and basal ganglia in a group of ID patients and such patterns was associated with cognitive decline in those patients (15).

The amygdala plays a key role in processing negative emotional arousal and fear-inducing stimuli and therefore is involved in the hyperarousal model of ID (14, 45). Gong et al. also reported atrophy in the left superficial and right basolateral nucleus of amygdala, the association of insomnia severity with shape of the right centromedial nucleus, and the link between anxiety and shape of the basolateral nucleus of amygdala (16). Beside structural changes, functional imaging studies demonstrated that the amygdala response to insomnia-related stimuli is more robust in ID than healthy controls with lower habituation (17). Furthermore, decreased functional connectivity between amygdala and insula, striatum, and thalamus, as well as increased functional connectivity of amygdala with premotor and sensorimotor cortex in ID have been reported (46). Impaired connectivity and dysfunction of the amygdala due to emotional processing is a shared phenomenon in major depressive disorder (MDD) and ID (9, 17, 46). It has been demonstrated that amygdala volume is larger in MDD patients with insomnia symptoms compared to MDD patients without insomnia, regardless of the depression subtype (e.g., melancholic or psychotic) (9, 47).

Similar to the current study, hippocampal atrophy was previously reported in patients with ID (13). It has been revealed that distinct connectivity patterns of anterior and posterior hippocampus involve in memory processing and encoding success (48, 49). A multimodal parcellations and behavioral decoding of hippocampal sub-regions demonstrated a head–body and tail partition, subdivided along the anterior–posterior and medial–lateral axis and behavioral analyses suggested an emotion–cognition gradient along the anterior–posterior axis (50). According to our results, major hippocampal abnormality was observed within the head and body of the hippocampus. Previously, Joo et al. found that patients with ID had bilateral atrophy in the body and tail of hippocampus (i.e., CA2 and DG), which was associated with impaired cognitive functions, as well as in the head of hippocampus (i.e., CA1), which is associated with poor sleep quality (51). Some studies identified disruption of hippocampal functional connectivity within the default mode network (DMN) in ID (52). In particular, enhanced functional connectivity between the retrosplenial cortex/hippocampus and different hubs of the DMN is reported in ID previously (52). CBT-I normalized DMN hyperactivity and improved symptoms and quality of life of patients with psychophysiological insomnia (53). A meta-analysis found that patients with ID consistently have poor daily performance in several cognitive functions including working memory, episodic memory, and problem solving, which may be related to hippocampal dysfunction in ID (54).

The figure of thalamus in sleep regulation is well-established as well (55, 56). The anterior and dorsomedial nuclei of thalamus are responsible for organization of wake-sleep pattern, and affect pineal melatonin production and secretion (57). Thalamic lesions cause severe and persistent insomnia in the animal models (57). Severe impairment in mitochondrial function, protein synthesis, and neuronal loss in mediodorsal thalamus has been observed in fatal familial insomnia (58). Few studies on ID demonstrated structural and functional abnormalities in the thalamus. For example, patients with ID showed thalamic atrophy, as well as disruption of thalamus's functional connectivity with the ACC, orbitofrontal cortex, hippocampus, caudate, and putamen which were negatively correlated with PSQI score (59). Kim et al. observed cortical and thalamic hyperactivity in response to sleep-related tasks in psychophysiological insomnia (60). These studies indicate maladaptive role of amygdala, hippocampus, and thalamus in pathophysiology of ID, mainly in psychophysiological subtype.

## Limitations

The results of this study should be interpreted with cautious, due to several potential limitations. Small sample size together with potential variability in shape analysis may over-estimate some structural alterations or miss some others. A stronger magnetic field e.g., 3T or 7T would increase signal to noise of the images and therefore increase the accuracy of subcortical segmentations. Moreover, in the current study, mean age of patients were higher than healthy subjects and it has been demonstrated that age has an important effect in gray matter structures in subjects with sleep disturbances (61, 62). Smaller total brain size could be argued to contribute in the group difference reported in this work. However, the effect of any total brain atrophy associated with normal aging would be diminished when age was taken as a covariate of no-interest, as applied in our analysis. In fact, total brain volume was lower in paradoxical than in psychophysiological insomnia patients. This argues that perhaps there is biological effect in paradoxical insomnia, which is associated with brain atrophy beyond what is normally seen in aging. Thus, although we included age as covariates of no-interest in all analyses, careful matching of the groups and controlling for the effects of comorbid anxiety and depression should be considered in the future studies. Combination of structural assessment with functional MRI and/or positron emission tomography (PET) using a multimodal approach could further enlighten the role of subcortical structures in pathophysiology of ID and its subtypes. Unfortunately, such tools were not available in our center at the time of study. Further longitudinal studies with high magnetic fields and molecular imaging techniques in a larger cohort are needed to endorse our findings.

## Conclusion

The present work demonstrated structural alterations in the amygdala, hippocampus, corpus striatum, and thalamus in ID. Shape alterations were prominent in the caudate, putamen, and nucleus accumbens in paradoxical insomnia and were noticeable in the thalamus, amygdala, and hippocampus in psychophysiological insomnia. The volume of caudate was different between ID patients and controls, as well as between ID subtypes. The structural changes are associated with subjective and objective sleep symptoms and support different neurobiological mechanisms between paradoxical and psychophysiological insomnia. This study highlights the need for classification of ID and may have a great impact on clinical trials and developing better treatment for ID in future. Clearly, we need global sharing of multimodal imaging-genetic data using world-wide initiatives like ENIGMA-Sleep (http://enigma.ini.usc.edu/ongoing/enigma-sleep/) in sleep medicine (63).

## Data Availability Statement

The raw data supporting the conclusions of this article will be made available by the authors, without undue reservation.

## Ethics Statement

The studies involving human participants were reviewed and approved by Kermanshah University of Medical Sciences. The patients/participants provided their written informed consent to participate in this study.

## Author Contributions

FE, MT, MK-A, SM, HK, and MZ contributed to design and conceptualization of the idea. FE, KN, and MR collected data. MM and MZ analyzed data. All authors drafted the manuscript and revised the manuscript for intellectual content.

## Conflict of Interest

The authors declare that the research was conducted in the absence of any commercial or financial relationships that could be construed as a potential conflict of interest.
